# Health Promoting Effects of Brassica-Derived Phytochemicals: From Chemopreventive and Anti-Inflammatory Activities to Epigenetic Regulation

**DOI:** 10.1155/2013/964539

**Published:** 2013-12-23

**Authors:** Anika Eva Wagner, Anna Maria Terschluesen, Gerald Rimbach

**Affiliations:** Institute of Human Nutrition and Food Science, Christian-Albrechts-University Kiel, Hermann-Rodewald-Stra**β**e 6, 24118 Kiel, Germany

## Abstract

A high intake of brassica vegetables may be associated with a decreased chronic disease risk. Health promoting effects of Brassicaceae have been partly attributed to glucosinolates and in particular to their hydrolyzation products including isothiocyanates. *In vitro* and *in vivo* studies suggest a chemopreventive activity of isothiocyanates through the redox-sensitive transcription factor Nrf2. Furthermore, studies in cultured cells, in laboratory rodents, and also in humans support an anti-inflammatory effect of brassica-derived phytochemicals. However, the underlying mechanisms of how these compounds mediate their health promoting effects are yet not fully understood. Recent findings suggest that brassica-derived compounds are regulators of epigenetic mechanisms. It has been shown that isothiocyanates may inhibit histone deacetylase transferases and DNA-methyltransferases in cultured cells. Only a few papers have dealt with the effect of brassica-derived compounds on epigenetic mechanisms in laboratory animals, whereas data in humans are currently lacking. The present review aims to summarize the current knowledge regarding the biological activities of brassica-derived phytochemicals regarding chemopreventive, anti-inflammatory, and epigenetic pathways.

## 1. Introduction

**Figure 1:**
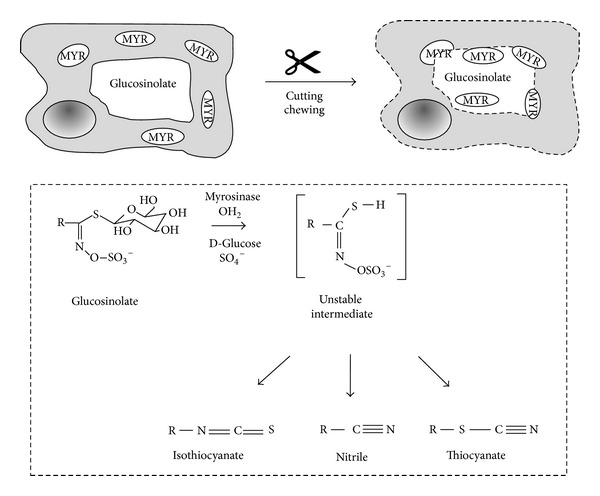
Myrosinase-mediated hydrolysis of glucosinolates with main break-down products (modified according to [114]).

Epidemiological studies link a high intake of brassica vegetables with a lower incidence for different kinds of cancers [1–3]. Health promoting effects of brassica vegetables have been attributed to glucosinolates, sulfur containing compounds almost exclusively present in plants of the family Brassicaceae. However, these chemopreventive effects are not mediated by glucosinolates *per se* but mainly through isothiocyanates, one of the major hydrolysis products resulting from myrosinase cleavage [4–9]. Myrosinase is a thioglucohydrolase located apart from glucosinolates in so-called myrosin cells. Upon plant cell disruption enzyme and glucosinolate get in contact and hydrolization is initiated. Based on reaction conditions (e.g., pH, temperature) either isothiocyanates, thiocyanates, or nitriles are formed (Figure 1) [10, 11].

**Figure 2:**
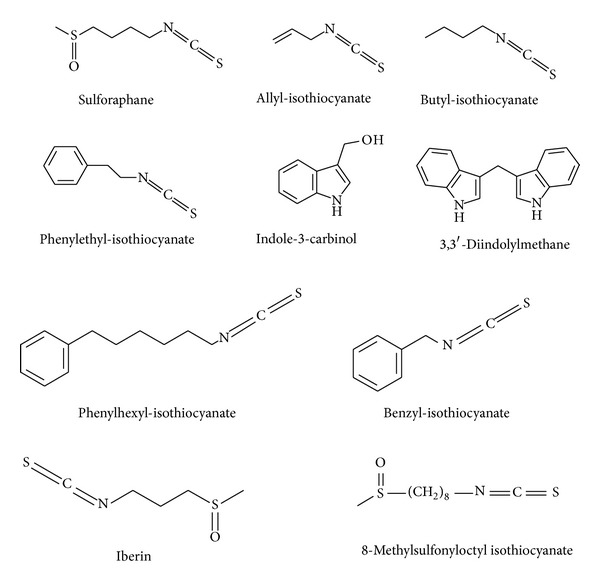
Chemical structures of selected aliphatic and aromatic brassica-derived phytochemicals.

Several *in vitro *and *in vivo* studies suggest that brassica derived phytochemicals may counteract inflammatory pathways and exhibit chemopreventive activity. Furthermore, impact of glucosinolates and/or their corresponding hydrolyzation products on epigenetic mechanisms including DNA-methylation, histone modification, and microRNAs has been recently described and is in the focus of the present paper. Chemical structures of selected brassica-derived phytochemicals are presented in Figure 2.

## 2. Brassica-Derived Phytochemicals Target Inflammatory Pathways

Several studies suggest anti-inflammatory properties of brassica-derived phytochemicals [12]. Besides others, these beneficial effects may be mediated through an induction of antioxidant and phase 1/2 genes and the inhibition of proinflammatory signaling pathways via regulation of various transcription factors which may be further controlled by epigenetic modifications and miRNAs [11, 13–15]. Additionally, it has been shown that brassica derived phytochemicals exhibit anti-infective and antiviral activity (e.g. inhibiting *Helicobacter pylori*) [16–18]. In this context the transcription factor nuclear factor kappa B (NF*κ*B) is a central player in inflammatory processes. In general, NF*κ*B resides inactively in the cytosol as a heterodimer consisting of two subunits, for example, p50 and p65, bound to its inhibitory protein I*κ*B*α* [19]. NF*κ*B can be activated by a wide variety of proinflammatory stimuli including cytokines and reactive oxygen species resulting in the activation of upstream kinases, phosphorylation, ubiquitination, and consequently the degradation of I*κ*B*α* [20]. Once p50 and p65 have been released, they translocate to the nucleus and bind to the *κ*B site located in the promoter regions of the DNA of target genes thereby driving gene expression [21, 22]. NF*κ*B target genes include, for example, cyclooxygenase 2 (COX-2), inducible nitric oxide synthase (iNOS) (inflammatory function), Bcl-Xl, Bcl-2, Bcl-3 (anti-apoptotic function), MYC (cell division), matrix metalloproteinase (MMP), and vascular endothelial growth factor (VEGF) (angiogenesis) [23–25]. Due to NF*κ*Bs central role in inflammation the transcription factor seems to be an attractive target to treat inflammation related diseases.

A variety of naturally occurring NF*κ*B inhibitors have been described including brassica derived phytochemicals like sulforaphane (SFN), phenethyl-isothiocyanate (PEITC), 8-methylsulphinyloctyl isothiocyanate (MSO), and indole-3-carbinol (I3C). They downregulate lipopolysaccharide (LPS) induced activation of NF*κ*B and suppress COX-2, iNOS, and prostaglandin (PG) expression in cultured mouse macrophages, possibly via inactivating NF*κ*B [26–31]. Furthermore, 3,3′-diindolylmethane (DIM) significantly reduced PGE(2), NO, proinflammatory cytokines, and the number of colon tumors in BALB/c mice suffering from colitis associated colon cancer [32]. In C57BL/6 mice we observed that a 7-day pretreatment with SFN resulted in less severe symptoms of DSS-induced colitis as compared to PBS-pretreated controls [33]. Interestingly, Prawan et al. (2009) have observed that synthetic ITCs show an even stronger anti-inflammatory activity than that of their natural counterparts [30]. However, the underlying mechanisms of how brassica derived phytochemicals modulate the NF*κ*B pathway are only partly understood. Most studies indicate that ITCs inhibit DNA binding of NF*κ*B, suppress its translocation, or stabilize I*κ*B*α* through a decreased phosphorylation of *Ικ*B kinase complexes (IKKs) [26, 30, 34–36]. This in turn suggests that ITCs target upstream factors like MAPK pathways (e.g., ERK, JNK, or p38) resulting in NF*κ*B inhibition [30]. A study conducted by Yang and coworkers (2010) confirmed this hypothesis by demonstrating that PEITC inhibits ERK1/2, MAPK kinase 7 (MAPKK7), and MAPK kinase kinase 3 (MAPKK3) [37]. ITCs may also act anti-inflammatorily through lowering ROS-induced NF*κ*B activity [34]. Heiss and coworkers observed that SFN impairs DNA-binding of NF*κ*B which was not accompanied by I*κ*B degradation and nuclear translocation of NF**κ**B [26]. It is supposed that SFN interacts with thiol groups, forms dithiocarbamates, and binds directly to redox-regulated cystein residues (Cys62 and Cys38) of the p50 and p65 subunits which prevent DNA binding [38]. A modification of these cystein residues may provide an alternative strategy for phytochemical mediated chemoprevention [20]. Brassica derived phytochemicals may also mediate anti-inflammatory effects through an interaction with reduced redox regulators like glutathione, thioredoxin, or redox factor 1 (ref-1), leading to changes of the reducing milieu required for correct DNA binding [39].

## 3. Brassica-Derived Phytochemicals Target Chemopreventive Pathways

Nrf2 is a transcription factor playing a crucial role in regulating inflammation and chemoprevention. Under basal conditions Nrf2 is bound to its cytosolic inhibitor, the Kelch like ECH-associated protein 1 (Keap1) [40]. In the presence of activating agents including isothiocyanates and other electrophiles Nrf2 may be activated through two distinct cellular signaling pathways resulting in the liberation of Nrf2 from its inhibitor Keap1. Nrf2 can either be phosphorylated through an activation of upstream protein kinases which causes the destruction of the Nrf2-Keap1 complex or in the presence of pro-oxidants the cysteine thiols of Keap1 may be modified which promotes dissociation of Nrf2 from Keap1. Liberated Nrf2 translocates to the nucleus where it binds together with several cofactors including small Maf proteins (MafF, MafG, and MafK), c-Jun, and cAMP response element-binding (CREB) protein (CBP) to the antioxidant response elements (AREs) in the promoter regions of genes encoding antioxidant and detoxifying phase II enzymes like NADPH quinone oxidoreductase 1 (NQO1), hemeoxygenase-1 (HO-1), superoxide dismutase (SOD), glutamyl cysteine ligase (GCL), and GST [20, 41]. Khor et al. (2006) have reported that Nrf2-deficient mice suffer under a more severe dextran-sulfate-sodium- (DSS-)induced colitis than Nrf2+ mice which was accompanied by a decreased expression of antioxidant and phase II enzymes and an increased level of proinflammatory mediators like COX-2, iNOS, interleukin 1*β* (IL-1*β*), and tumor necrosis factor *α* (TNF*α*) [42]. Different phytochemicals can lead to nuclear accumulation of Nrf2 [43, 44]. In particular, the effect of SFN on Nrf2 pathways has been intensively investigated, showing that SFN *in vitro *and in animal studies successfully activates phase 2 and antioxidant enzymes via induction of Nrf2 [45–48]. Also other ITCs such as allyl-isothiocyanate (AITC), butyl-isothiocyanate (BITC), and PEITC have been shown to exhibit similar Nrf2 inducing activity *in vitro *[48, 49]. PEITC has been proven to be an even stronger inducer of Nrf2-ARE mediated signaling pathways than SFN [50].

There is evidence that overexpression of Nrf2 can modulate NF*κ*B expression, since NF*κ*B competes with Nrf2 for binding to the transcriptional coactivator CREB-binding protein [51, 52]. Accordingly, Surh and Na described a cross-talk between NF*κ*B and Nrf2 signaling supported by the fact that most of the phytochemicals exhibit both, anti-inflammatory and anti-oxidant properties [20].

## 4. Brassica Derived Phytochemicals Target Epigenetic Pathways

Epigenetics describe a heritable change in gene expression that is not mediated through a change of DNA-sequence [53]. Epigenetic changes comprise DNA-methylation, histone modifications, and microRNA expressions [53–55]. Several studies suggest that brassica derived phytochemicals affect epigenetic mechanisms. Epigenetic aberrations take place in the early stages of carcinogenesis and partly represent an initiating process of cancer development. Phytochemicals may intervene in this process and incarnate potential targets for cancer prevention [56]. Posttranslational modifications at the N-terminal tail of histones are one epigenetic mechanism that plays a role in gene regulation and carcinogenesis [56, 57]. Histones can be post-translationally modified by acetylation, deacetylation, phosphorylation, ubiquitinylation, sumoylation, and ADP ribosylation [58, 59]. Biotinylation has also been suggested to mediate histone modification. However, recent studies conducted by Li and coworkers as well as by Xue and colleagues discovered that it is not a biotinylation *per se* but an assembly of different proteins including biotin ligase and holocarboxylase synthetase interceding histone modification [60, 61]. Histone acetylation and deacetylation are the most analysed modifications mediated through a coaction of histone acetyl transferases (HAT) and histone deacetylases (HDAC) [56] resulting in gene activation and inhibition of gene activity, respectively [57, 62]. HATs transfer acetyl groups from acetyl-CoA onto lysine residues at the histone [63] while HDACs detach histone acetyl group transferring them onto CoA [56]. Chromatin acetylation by HATs opens the chromatin structure providing a possibility for transcription factors to approach the DNA which may lead to gene activation [57]. HATs are divided into four families on the basis of their structure homologues. At present, however, there is no literature data available presenting effects of brassica derived phytochemicals on HAT activity. Several studies suggest an effect of these phytochemicals on HDACs. HDACs are also divided into four groups according to their structure homology to yeast deacetylases [64, 65]. Both, *in vitro *and *in vivo *studies present ITCs as potent HDAC inhibitors [66]. Recent investigations by Rajendran and coworkers [67, 68] revealed a dose-dependent inhibition of HDAC activity and an increase of HDAC protein turnover following ITC incubation of HCT116 colon cancer cells that was proportionally dependent on their alkyl chain length. Besides colon cancer cells, HDAC inhibitory effects of SFN have been shown in various prostate epithelial cells—normal prostate epithelial cells (PrEC), benign hyperplasia (BPH1), and cancerous (LnCaP, PC-3) prostate epithelial cells [69, 70] as well as in different breast cancer cells [71]. The HDAC inhibitory effect of SFN has also been confirmed in an *in vivo* model [72, 73]. Also other ITCs, including PEITC [74], the synthetic phenylhexyl-ITC (PHI) [75–77] and benzyl-ITC (BITC) [78], inhibit HDAC activity in different cell lines. The indole DIM, a known brassica derived plant bioactive, has been reported to decrease HDAC activity in the prostate cancer cell lines PC-3 and LnCaP. Interestingly, the monomer of the compound I3C had only a weak effect on LnCaP cells, which are androgen sensitive, and non on PC-3 cells being androgen-insensitive [79]. In a clinical trial it turned out that SFN-rich broccoli sprouts consumed by healthy human subjects exhibit HDAC inhibitory effects [73, 80].

**Figure 3:**
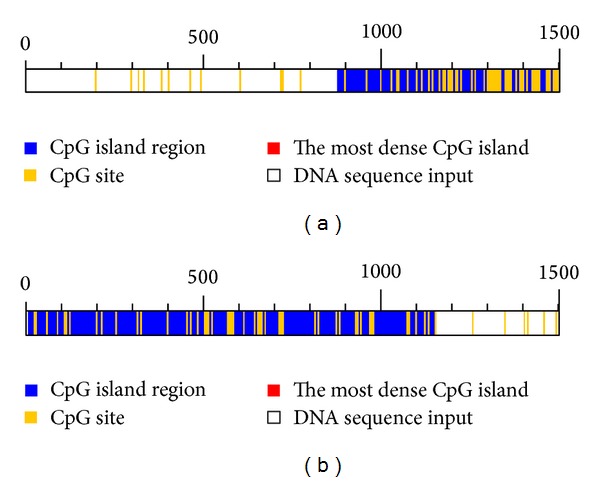
CpG islands in the promoter of Nrf2 (a) and Keap1 (b) identified by using the database *dbcat* (http://dbcat.cgm.ntu.edu.tw/).

DNA methylation is regulated by various DNA-methyltransferases (DNMT 1, 3a, 3b) that transfer methyl groups from the methyl precursor S-adenosyl-L-methionine (SAM) to the C5-position of certain cytosines and influence gene transcription [81, 82]. Gene promoter regions rich in CpG sequences—so called CpG islands—are generally unmethylated in normal cells [56], whereas transformed cells often show hypermethylated promoters and/or genome wide hypomethylation [57]. Predicted CpG islands in the promoters of Nrf2 and Keap1 are shown in Figure 3. Besides carcinogenesis changes in DNA methylation patterns are also associated with ageing and the development of chronic degenerative diseases [56, 83]. Currently only little information regarding DNMT inhibition by ITCs is available in the literature. The main investigated ITC regarding its DNMT inhibiting activity is SFN. Studies conducted by Meeran and colleagues [84] in the human breast cancer cell lines, MCF-7 and MDA-MB-231, revealed an inhibiting effect of SFN on DNMT1 and DNMT3a. SFN has been reported to inhibit DNMT-expression in the human colorectal cancer cell line CaCo-2 [85], in LnCaP human prostate cancer cells [86], and in porcine satellite cells [87]. Furthermore, effects of the naturally occuring ITC iberin [85] and the synthetic ITC PHI on DNMT inhibition in colorectal cancer cells and in the acute lymphoid leukemia cell line Molt-4 [88] have been described. However, *in vivo* data for DNMT inhibition by brassica derived phytochemicals is lacking and needs further investigation.

**Figure 4:**
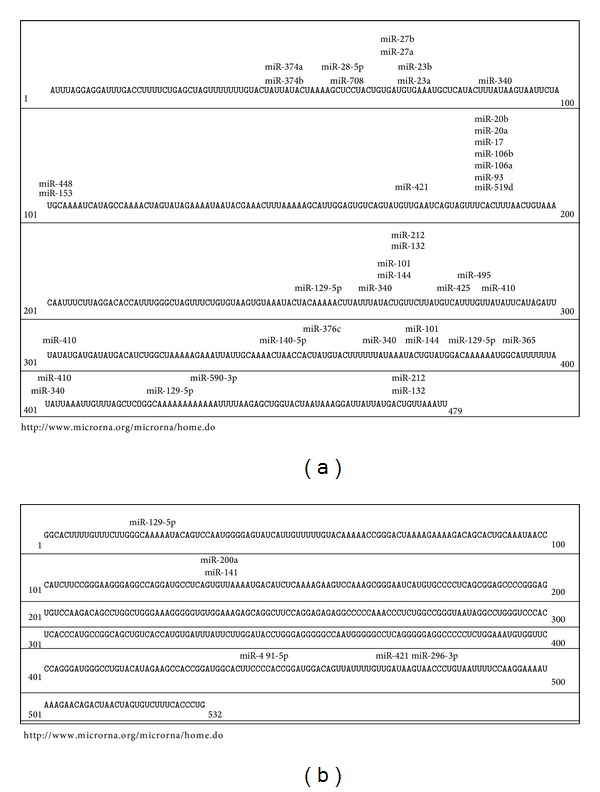
Predicted binding sites of conserved microRNAs of Nrf2 (a) and Keap1 (b) as identified by http://www.microrna.org/microrna/home.do.

MicroRNAs (miRNA) represent a class of evolutionary conserved small noncoding RNAs with a length of ~22 nucleotides that control gene expression at the posttranscriptional level [89–91]. MicroRNAs bind to the 3′ untranslated region (3′UTR) of target mRNAs and lead, depending on the base pair complementarity between microRNA and target mRNA, to a decreased translation and mRNA degradation, respectively [92, 93]. MicroRNAs are involved in a number of several cellular processes in the organism including development, cell-proliferation, differentiation, and apoptosis [91, 94, 95]. There are some data pointing to the notion that Brassica derived phytochemicals modulate miRNA expression suggesting that this is one mechanism of how these compounds may mediate health promoting activity. Slaby and coworkers [96] treated the colonic epithelial cell lines NCM460 and NCM356 with the isothiocyanates iberin and SFN which resulted in the regulation of three common microRNAs—miR-155, miR-23b, and miR-27b. Interestingly, it has been found that PEITC affects miR-141 in LnCaP cells, a microRNA which has been closely linked to the development of prostate cancer [97]. Also Basu and colleagues [98] observed microRNA-modulating effects according to isothiocyanate treatment of mice suffering from pancreatic intraepithelial neoplasia. The authors identified an inhibition of miR-221 and miR-375 following benzyl-ITC treatment changing hyperproliferative into hypoproliferative pancreatic cancer cells. Our own studies revealed an inhibitory effect of allyl-isothiocyanate (AITC) and SFN on the proinflammatory and oncogenic miR-155 in RAW264.7 murine macrophages [99]. In the lungs of mice exposed to environmental cigarette smoke microRNA expression has been altered which could be counteracted by PEITC treatment. However, some side effects of PEITC, including dysregulation of hepatic microRNAs, have been observed which should be investigated in more detail [100, 101]. Furthermore, the brassica derived phytochemical I3C has been shown to reverse vinyl-carbamate induced de-regulation of microRNAs and the I3C dimer DIM increased miR-146a expression which in turn resulted in a decrease of cell invasion [102, 103]. To predict microRNA binding sites in the 3′UTR of different genes internet based prediction tools, including http://www.microrna.org/microrna/home.do, are available. According to http://www.microrna.org/microrna/home.do more than 40 conserved microRNAs may bind to Nrf2 (Figure 4(a)). However, only a small number of microRNAs, including miR-28, miR-93, miR-144, miR-153, miR-27a, miR-132, and miR142-5p, have been confirmed experimentally to directly bind to the 3′UTR of Nrf2 and consequently downregulate gene expression [104–108]. Most of the microRNAs discussed in the literature to impact Nrf2 are affecting the expression of the transcription factor indirectly. Eades and coworkers [109] observed that miR-200a degrades Keap1 mRNA which in turn causes an increase of Nrf2 levels in breast cancer cells. A recent study conducted by Petrelli and colleagues [110] confirmed the effect of miR-200a in preneoplastic lesions, where miR-200a also lowered Keap1 expression resulting in an Nrf2 increase. Also miR-141 binds to Keap1 which results in increased Nrf2 levels [111]. http://www.microrna.org/microrna/home.do suggests five conserved microRNAs to bind to Keap1 from which already two have been confirmed to effectively target the 3′UTR of Keap1 (Figure 4(b)).

**Figure 5:**
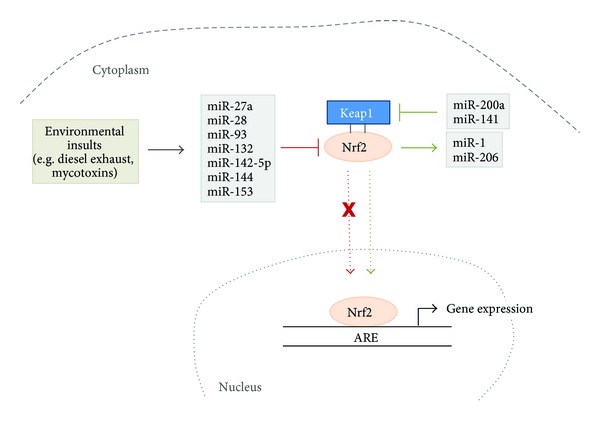
MicroRNAs being involved in the regulation of Nrf2 and Keap1.

The expression of Nrf2 regulating microRNAs is modulated by several environmental insults including diesel exhaust [112] and the mycotoxin ochratoxin A [108]. Interestingly, a study conducted by Singh and coworkers suggests that Nrf2 itself targets microRNAs. The authors observed an increase of miR-1 and miR-206 levels while Nrf2 is simultaneously upregulated [113].  Figure 5 gives an overview of how microRNAs are involved in Nrf2/Keap1 signaling.  Table 1 lists confirmed microRNAs that target the promoter region of Nrf2 and Keap1.

## 5. Summary and Conclusion

**Figure 6:**
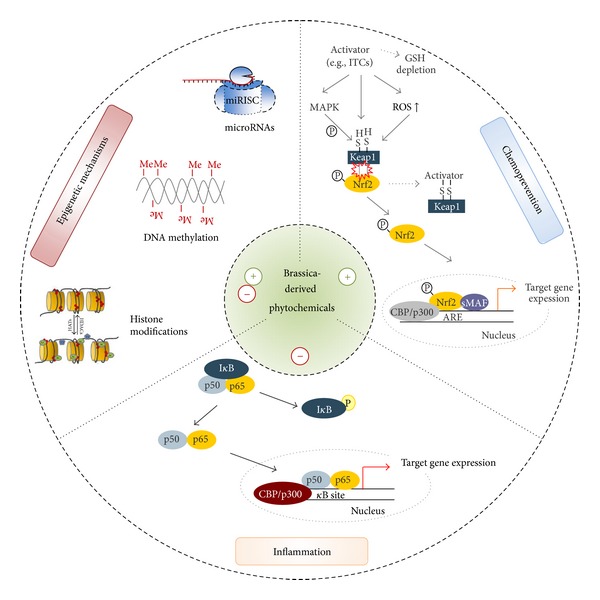
Potential chemopreventive, anti-inflammatory and epigenetic mechanisms by which brassica-derived phytochemicals may mediate health benefits (partly adapted from [11, 115–118]).

ITCs are one of the major degradation products of glucosinolates derived from brassica vegetables and are partly held to be responsible for the health promoting effects observed in populations with a high consumption of Brassicaceae. The health promoting effects of a diet rich in Brassica vegetables have been known for several decades and seem to be a promising starting point for, for example, the development of chemopreventive and anti-inflammatory functional foods, dietary supplements, and drugs.  Figure 6 gives an overview of how brassica-derived phytochemicals may mediate their health-promoting activity. An increasing number of studies deal with ITC effects on the chemopreventive transcription factor Nrf2 and its corresponding target genes including antioxidant and phase 2 enzymes. In addition to studies in cultured cells beneficial effects of ITC have also been confirmed *in vivo* in laboratory rodents and humans. Also the anti-inflammatory effects of ITCs are well documented in both cell culture and *in vivo* studies. The applied concentrations of brassica derived phytochemicals range between 0.25 and 100 *μ*mol/L in *in vitro *studies and up to 90 mg/kg BW in mouse studies. However, with regard to ITC effects on epigenetic mechanisms including histone modifications, DNA methylation, and microRNAs only a limited number of studies are currently available. In this context several studies have been performed in cultured cells and have revealed an increase in epigenetic targets connected with an inhibition of HDAC and DNMT as well as on microRNA expression. However, data concerning the modulation of epigenetic targets by ITCs especially in humans are, by and large, lacking even though epigenetic targets seem to play an essential role in the development of different kinds of cancer and may depict a promising target to prevent and/or treat chronic diseases.

## Figures and Tables

**Table 1:** MicroRNAs inhibiting transcription factor Nrf2 and its inhibitor protein Keap1.

MicroRNA targeting Nrf2	Research model	Reference
miR-27a	SH-SY5Y cells	[105]
miR-28	MCF-7 cells	[104]
miR-93	MCF-10A cells T47D cells ACI rats	[107]
miR-132	LLC-PK1 cells	[108]
miR-142-5p	SH-SY5Y cells	[105]
miR-144	K562 cells	[106]
miR-153	SH-SY5Y cells	[105]

MicroRNA targeting Keap1	Research model	Reference

miR-141	A2780 cells TOV112D cells TPV21G cells	[111]
miR-200a	MDA-MB-231 cells Hs578T cells MCF-10A	[109]
